# Supplementary material for: Interruptions in a dental setting and evaluating the efficacy of an intervention: A pilot study
Source: PLoS One. 2024 Apr 4;19(4):e0296677. doi: 10.1371/journal.pone.0296677 (PMC10994382; doi:10.1371/journal.pone.0296677)
Supplement: Attachment — Submitted filename: Response to Reviewers.docx [file pone.0296677.s001.docx]

**Response to Reviewer Comments on**

***An Observational Study of Interruptions in a Dental Setting***

***and Evaluating the Efficacy of an Intervention***

**We appreciate the time and effort spent by the reviewers on our submission. We are glad to learn that the reviewers and the editor are appreciative of the relevance and contribution of our work. Response to each reviewer comments is highlighted in BOLD below. The corresponding revisions are reflected in BOLD in the revised manuscript.**

**Reviewer #1**

The article discuss the interruptions during dental procedures and its impact on the provider. The research question wasn't stated clearly in a sense what is the hypothesis?

**RE: We would like to clarify that our research was exploratory in nature, not hypothesis-driven. While we did know that interruptions were affecting the operations at this dental clinic, without this exploratory work, we would not have known what caused such interruptions, nor would we have known what type of intervention to design and implement. Such studies are typical in the research area around interruptions. Hope this clarifies this issue. We have included the above briefly in the Introduction section.**

1. It is causal that when interruptions occur during any given procedure will lead to delays and negative impacts. using the switchable signs reduced the interruptions as the statistics showed, however, the technical factors were missing from the collected data, for example What time of the day most of the interruptions happening, What type of procedure was conducting (intensity of the procedure and the focus varies).

**RE: About the ‘time of the day,’ we acknowledge that this was not conveyed properly in our original submission. During editing, Figures 7 and 8 were swapped and the caption of our Figure 7 was lost, and we take the responsibility for that. That specific figure shows the distribution of interruptions over the course of the day, now numbered Figure 7. Hope that addresses your query.**

**About the ‘type of procedure,’ during data collection, we kept track of the general type of treatment that was performed, but did not record every sub-step that might have been affected. As far as provider satisfaction was concerned, any interruption that had providers move their eyes away from the tooth they were working on was dissatisfactory. This is because refocusing their eyes was considered an unnecessary discomfort as the light conditions change drastically (dental lighting can have 50.000lux+) and the parts they focus on are relatively small, of a similar color, and they will only see properly within a small distance range. Hope this addresses your inquiry. We now add this succinctly in Section 2 (Methods / Data Collection) of the revised manuscript.**

2. Plus other interruptions like calls/intercom from the reception weren't measured. The research paper measured on type of interruptin and tested the switch sign neglecting other interruptions variable in the dental clinic setting.

**RE: We apologize that we did not clarify in the Settings that the infrastructure of the clinic had originally been set up without phones in treatment rooms or an intercom system to avoid interruptions and distractions during treatments. All interruptions had to pass through the door to the respective treatment room. We add this clarification in the Settings section of the revised manuscript.**

**Reviewer #2**

Thank you for this interesting, pertinent and well-written paper. Several comments related to the methods (data analysis):

I am interested to know why a test of significance was not applied, applying a relevant test will make the findings observed differences) more meaningful. For instance, the authors may look for the mean differences in the duration of the interruptions before and after. I see that authors have shown the differences the duration in figure 10. However it would be worthwhile to apply an appropriate test if significance, rather than only looking at differences.

**RE: *That is a good point. We now analyze the interruptions by conducting a pairwise comparison of interruptions by duration before and after the intervention (previously illustrated through Figure 10). This is now summarized in the Results section.***

1. 1. Regarding the ‘qualitative data’. Will it be appropriate to call a survey with likert scale responses as qualitative data? Did the authors employ qualitative data collection methods? The findings/providers responses from the ‘survey’ have been quantified (table 2, 3 and 4). I would suggest rephrasing this heading and avoid using the term ‘qualitative data’.

**RE: That is a good point. We conducted a survey and measured the data on a Likert Scale, which was numeric. So an accurate way would be to replace ‘qualitative’ with ‘quantitative survey.’ We have made this change throughout the manuscript.**

2. It will be useful to clearly define ‘critical procedures’ for the readers.

**RE: Your point is well-taken, and we acknowledge that some readers might be interested in more details. As this paper is not focused on the medical aspects in dentistry, we left the definition of what should be considered a critical procedure up to the providers. These included – for example – work close to the pulp, endodontic treatments close to the apex, certain steps during adhesive attachment and the like. In general, anything that had a significantly higher chance of harming the patient or creating disproportionate rework effort was considered critical. The observer clarified his relevant observations with the provider to make sure these were properly identified after the patient left. We now succinctly state this in Section 2 Methods /Data Collection /Pre-intervention period of the revised manuscript.**

3. Minor comments:
   Below are some of the typos but the whole manuscript needs to be proof read.(particularly for tenses)
   Please spell out (CEREC) in the introduction.
   P.2. To address these questions, we use actual data collected at a German dental clinic…. (To address these questions, we ‘used’ actual data collected at a German dental clinic
   P.5. The remaining 15% (28/194) fall in other categories (mainly technical issues). (‘fell’ in other category?)

**RE: We have proof-read the manuscript and fixed all tense issues. We now use past tense throughout the manuscript and have revised the sentences per your P.2 and P.5 comments.**

**Instead of CEREC, which is a trademark, we now use *dental chairside CAD/CAM system*.**

**Reviewer #3**

This is an interesting study.

1.The sample size limitation dictates that the findings/results cannot be generalized.

**RE: We concur with your assessment and clearly state this in the Conclusion section.**

2. I would suggest adding, A pilot study to the title.

**RE: We appreciate your suggestion. Our new title now reads: *Interruptions in a Dental Setting and Evaluating the Efficacy of an Intervention: A Pilot Study***

3. References should be completed and as per guidelines to the authors

**RE: We are not sure what this comment refers to. All references were checked and they are complete. If there is any missing information, then please point to the reference # and we would be happy to add missing details.**
